# Whole-Genome Sequence of Muju Virus, an Arvicolid Rodent-Borne Hantavirus

**DOI:** 10.1128/genomeA.00099-14

**Published:** 2014-02-27

**Authors:** Jin Goo Lee, Se Hun Gu, Luck Ju Baek, Ok Sarah Shin, Heung-Chul Kim, Terry A. Klein, Richard Yanagihara, Jin-Won Song

**Affiliations:** a Department of Microbiology, College of Medicine, and the Institute for Viral Diseases, Korea University, Seoul, Republic of Korea; b Department of Biomedical Science, College of Medicine, Korea University, Seoul, Republic of Korea; c 5th Medical Detachment, 168th Multifunctional Medical Battalion, U.S. Army 65th Medical Brigade, Unit 15247, Seoul, Republic of Korea; d Public Health Command Region-Pacific, Camp Zama, Japan; e Pacific Center for Emerging Infectious Diseases Research, John A. Burns School of Medicine, University of Hawaii at Manoa, Honolulu, Hawaii, USA

## Abstract

The complete genome sequence of Muju virus was determined from lung tissue samples of three royal voles (*Myodes regulus*) captured in Gangwon province in the Republic of Korea. Since few whole genome sequences of hantaviruses are available, this sequence may help to clarify the molecular phylogeny of arvicolid rodent-borne hantaviruses.

## GENOME ANNOUNCEMENT

Muju virus (MUJV), originally identified in the royal vole (*Myodes regulus*), an arvicolid rodent species that is endemic to South Korea, belongs to the *Hantavirus* genus of the *Bunyaviridae* family (1). Hantaviruses possess a negative-sense, single-stranded tripartite RNA genome, consisting of large (L), medium (M), and small (S) segments, which encode an RNA-dependent RNA polymerase (RdRp), envelope glycoproteins (Gn and Gc), and a nucleocapsid (N) protein, respectively (2). Lacking arthropod hosts, all hantaviruses are harbored by small mammals belonging to three taxonomic orders, namely, Rodentia, Soricomorpha, and Chiroptera (3). Some rodent-borne hantaviruses have been etiologically associated with diseases of varying clinical severity in humans known as hemorrhagic fever with renal syndrome and hantavirus cardiopulmonary syndrome (4). Partial sequences of one or more genomic segments are available for many hantaviruses, but comparatively few whole genome sequences have been deposited in GenBank. Given the sequence similarities of the partial MUJV genome with a pathogenic hantavirus known as Puumala virus, efforts were undertaken to obtain the complete genome sequence of MUJV.

Lung tissue samples were collected aseptically from royal voles captured using Sherman traps (H. B. Sherman, Tallahassee, FL) in Gangwon and Gyeonggi provinces in South Korea during 2011. All trapping and experimental procedures were approved by the Korea University Institutional Animal Care and Use Committee. Total RNA was extracted from 50 to 100 mg of lung tissue sample using RNA-Bee solution (Tel-Test, Inc., Friendswood, TX), and cDNA was synthesized using Moloney murine leukemia virus (M-MLV) reverse transcriptase (Promega, Madison, WI) with a highly conserved primer (5′-TAGTAGTAGACTCC-3′) and random hexamers. The 5′- and 3′-termini of each segment were amplified using the 3′-Full RACE Core set (TaKaRa Bio, Inc., Japan). Oligonucleotide primers, usually comprising 18 to 22 bp with a G+C content of 40 to 60%, were designed using the MegAlign Clustal W program (DNAStar, Inc., Madison, WI). For taxonomic verification of the rodent host, the 1,140-nucleotide mitochondrial DNA (mtDNA) cytochrome *b* gene was amplified by PCR, using previously described universal primers (5).

The full-length L genomic segment of MUJV strain 11-1 from Inje county and strains 11-4 and 11-5 from Pyeongchang county in Gangwon province was 6,544 nucleotides, with a predicted RNA-dependent RNA polymerase of 2,154 amino acids, starting at nucleotide position 37 and including 43 nucleotides of the 3′-noncoding region. The entire M genomic segment of the MUJV strains was 3,652 nucleotides, with a predicted glycoprotein precursor (GnGc) of 1,148 amino acids. The highly conserved WAASA amino acid motif of the M segment was found at amino acid positions 654 to 658. The full-length S segment of the MUJV strains from three royal voles was 1,831 nucleotides, with a predicted nucleocapsid protein of 433 amino acids. Based on full-length genomes, the sequence similarity among MUJV strains 11-1, 11-4, and 11-5 was 98.3 to 99.7% and 99.8 to 100% at the nucleotide and amino acid levels, respectively.

### Nucleotide sequence accession numbers.

The GenBank accession no. for the segments of the MUJV genome sequences are JX028271 to JX028273 for strain 11-1, JX046482 to JX046484 for strain 11-4, and JX046485 to JX046487 for strain 11-5.
